# Similar outcomes between adenoid cystic carcinoma of the breast and invasive ductal carcinoma: a population-based study from the SEER 18 database

**DOI:** 10.18632/oncotarget.14052

**Published:** 2016-12-20

**Authors:** Qing-Xia Chen, Jun-Jing Li, Xiao-Xiao Wang, Pei-Yang Lin, Jie Zhang, Chuan-Gui Song, Zhi-Ming Shao

**Affiliations:** ^1^ Department of Breast Surgery, Affiliated Union Hospital, Fujian Medical University, Fuzhou, China; ^2^ Department of Breast Surgery, Key Laboratory of Breast Cancer, Fudan University Shanghai Cancer Center, Shanghai Medical College, Fudan University, Shanghai, China

**Keywords:** adenoid cystic carcinoma, invasive ductal carcinoma, breast cancer-specific survival, overall survival

## Abstract

Adenoid cystic carcinoma of the breast (breast-ACC) is a rare and indolent tumor with a good prognosis despite its triple-negative status. However, we observed different outcomes in the present study. Utilizing the Surveillance, Epidemiology, and End Results (SEER) database, we enrolled a total of 89,937 eligible patients with an estimated 86 breast-ACC cases and 89,851 invasive ductal carcinoma (IDC) patients. In our study, breast-ACC among women presented with a higher proportion of triple-negative breast cancer (TNBC), which was more likely to feature well-differentiated tumors, rare regional lymph node involvement and greater application of breast-conserving surgery (BCS). Kaplan-Meier analysis revealed that patients with breast-ACC and breast-IDC patients had similar breast cancer-specific survival (BCSS) and overall survival (OS). Moreover, using the propensity score matching method, no significant difference in survival was observed in matched pairs of breast-ACC and breast-IDC patients. Additionally, BCSS and OS did not differ significantly between TNBC-ACC and TNBC-IDC after matching patients for age, tumor size, and nodal status. Further subgroup analysis of molecular subtype indicated improved survival in breast-ACC patients with hormone receptor-positive and human epidermal growth factor receptor 2-negative (HR+/Her2-) tumors compared to IDC patients with HR+/Her2- tumors. However, the survival of ACC-TNBC and IDC-TNBC patients was similar. In conclusion, ACCs have an indolent clinical course and result in similar outcomes compared to IDC. Understanding these clinical characteristics and outcomes will endow doctors with evidence to provide the same intensive treatment for ACC-TNBC as for IDC-TNBC and lead to more individualized and tailored therapies for breast-ACC patients.

## INTRODUCTION

Invasive breast cancers are a heterogeneous group of tumors that exhibit wide variation in their clinical presentation, behavior, and morphological spectrum [1]. Clinicians have designed different treatment plans for patients based on predictive and prognostic factors. To treat patients with heterogeneous cancer, it is critical to understand the specific biological characteristics associated with the prognosis and outcomes of a given histological type. Approximately 83% of invasive breast cancers are classified as invasive ductal carcinoma, not otherwise specified (IDC-NOS) [2, 3], whereas approximately 0.1%~1% are defined as adenoid cystic carcinoma (ACC) [1, 3]. Based on its clinicopathological characteristics and outcomes, ACC is distinct from IDC-NOS.

ACC is a rare malignancy of exocrine glands defined by the presence of a dual population of cells, and identical tumors can also arise from the breast. The unique characteristics of breast-ACC include a lack of expression of the estrogen receptor (ER), the progesterone receptor (PR), and HER2 and a basal-like phenotype in transcriptomic analysis [1, 4, 5]. However, HR+ breast-ACC has been reported [6, 7]. In contrast to the poor prognosis associated with other triple-negative breast cancers (TNBCs), ACC has been reported to exhibit a favorable prognosis and less aggressive behavior [8, 9], including a predominance among females and whites, a high percentage of low-grade tumors, localized stage tumors, absence of regional lymph node involvement, greater use of breast-conserving surgery (BCS), and lower use of chemotherapy [8, 9]. The 5-year overall and disease-free survival rates of breast-ACC are 94% and 82%, respectively [10]. However, ACC of the breast has been assigned a poor outcome based on a 70-gene poor prognosis profile and 21-gene high-risk recurrence score [1, 11, 12].

Due to its rarity, there are currently no established guidelines for treating this type of cancer, and there are large variations in patterns of practice. With respect to survival, BCS including postoperative radiotherapy (RT) appears to be equivalent to mastectomy alone [10, 13]. However, a Rare Cancer Network study reported that postoperative RT improved 5-year locoregional control (LRC) rates from 83% to 95% and that BCS is the treatment of choice for patients with ACC breast cancer [10]. The literature has increasingly recommended that BCS should be considered for ACC unless the tumor is large or the axillary lymph nodes are involved [14]. Accordingly, recent studies have reported a higher rate of patients treated with lumpectomy [6, 14, 15].

The identification of prognostic factors might enable more precise therapies for ACC patients. However, the effects of molecular subtype have not been investigated thoroughly in large population-based studies. Based on HER2 status recorded in the SEER database after 2010, the present study is the first to specifically provide insight into the effects of molecular subtype on breast-ACC outcomes compared to invasive ductal carcinoma of the breast (breast-IDC). Long-term survival has been calculated in the SEER database, with 5-year, 10-year, and 15-year relative survivals of 98.1%, 94.9%, and 91.4%, respectively [8]. Because an early peak of recurrence for the TNBC subtype occurs within the first 2–3 years after diagnosis, we conducted a short-term survival comparison between breast-ACC and breast-IDC, similar to a previous study of medullary breast carcinoma [16] and invasive cribriform carcinoma [17], and aimed to identify the differences in characteristics and outcomes between ACC and IDC with a large population-based dataset. Surprisingly, our conclusions based on the comparison of ACC and IDC are distinct from those of previous studies.

## RESULTS

### Demographics and clinical characteristics of the study population

Overall, 89,937 eligible patients were enrolled in our study, including 86 cases of breast-ACC and 89,851 cases of breast-IDC. The median follow-up time was 22 months. The baseline characteristics of the breast-ACC and breast-IDC subtypes are summarized in Table 1. There were significant differences in characteristics between the two subtypes, including grade, American Joint Committee on Cancer (AJCC) stage, tumor size, nodal status, breast subtype and type of surgery. Breast-ACC patients presented a higher proportion of TNBC (77.9% vs. 12.8%, *P* < 0.001), lower grade (grade I, 54.7% vs. 21.5%, *P* < 0.001), earlier stage (AJCC stage III, 0.0% vs. 9.0%, *P* = 0.007), and lower likelihood of nodal involvement (97.7% vs. 69.9%, *P* < 0.001). In addition, breast-ACC patients were more inclined to accept BCS than IDC patients (77.9% vs. 60.8, *P* = 0.001). Other tumor characteristics, including age, race, marital status, laterality, tumor size and radiation therapy, were similarly distributed between the two histological types.

**Table 1:** Baseline characteristics of patients with adenoid cystic carcinoma and invasive ductal carcinoma

Characteristics		ACC (*n* = 86)		IDC (*n* = 89,851)		Total (*n* = 89,937)		*P*^c^
		No	%	No	%	No	%	
**Median follow-up (months) (IQR)**		18 (9–31)		22 (10–34)		22 (10–34)		
**Age (years)**	**20–49**	22	25.6	23,849	26.5	23,871	26.5	0.840
	**50–79**	64	74.4	66,002	73.5	66,066	73.5	
**Race**	**White**	72	83.7	70,911	78.9	70,983	78.9	0.112
	**Black**	11	12.8	9,739	10.8	9,750	10.8	
	**Other^a^**	3	3.5	9,201	10.2	9,204	10.2	
**Marital status**	**Married**	58	67.4	56,258	62.6	56,316	62.6	0.355
	**Not married^b^**	28	32.6	33,593	37.4	33,621	37.4	
**Laterality**	**Left**	39	45.3	45,455	50.6	45,494	50.6	0.331
	**Right**	47	54.7	44,396	49.4	44,443	49.4	
**Grade**	**I**	47	54.7	19,315	21.5	19,362	21.5	**< 0.001**
	**II**	29	33.7	37,257	41.5	37,286	41.5	
	**III and IV**	10	11.6	33,279	37.0	33,289	37.0	
**AJCC stage**	**I**	48	55.8	50,293	56.0	50,341	56.0	**0.007**
	**II**	38	44.2	31,460	35.0	31,498	35.0	
	**III**	0	0.0	8,098	9.0	8,098	9.0	
**Tumor size (cm)**	**≤ 2**	49	57.0	58,548	65.2	45,494	65.2	0.093
	> **2 and ≤ 5**	35	40.7	27,316	30.4	44,443	30.4	
	**> 5**	2	2.3	3,987	4.4	3,989	4.4	
**Nodal status**	**0**	84	97.7	62,839	69.9	62,923	70.0	**< 0.001**
	**1 to 3**	2	2.3	20,567	22.9	20,569	22.9	
	**4 to 10**	0	0.0	4,430	4.9	4,430	4.9	
	**>10**	0	0.0	2,015	2.2	2,015	2.2	
**Breast subtype**	**HR+/Her2-**	17	19.8	63,667	70.9	63,684	70.8	**< 0.001**
	**HR+/Her2+**	0	0.0	10,271	11.4	10,271	11.4	
	**HR-/Her2+**	2	2.3	4,379	4.9	4,381	4.9	
	**Triple negative**	67	77.9	11,534	12.8	11,601	12.9	
**Type of surgery**	**BCS**	67	77.9	54,647	60.8	54,714	60.8	**0.001**
	**Mastectomy**	19	22.1	35,204	39.2	35,223	39.2	
**Radiation**	**No**	35	40.7	36,183	40.3	36,218	40.3	0.936
	**Yes**	51	59.3	53,668	59.7	53,719	59.7	

Abbreviations: ACC, adenoid cystic carcinoma; IDC, invasive ductal carcinoma; AJCC, American Joint Committee on Cancer; Her2, human epidermal growth factor receptor 2; HR, hormone receptor; BCS, breast-conserving surgery; IQR, interquartile range.

^a^Other includes American Indian/Alaskan native and Asian/Pacific Islander.

^b^Not married includes divorced, separated, single (never married), unmarried or domestic partner, and widowed.

^c^The *P* value of the Chi-square test was calculated between the ACC and IDC groups, and bold type indicates significance.

### Comparison of survival between breast-ACC and breast-IDC

**Figure 1:**
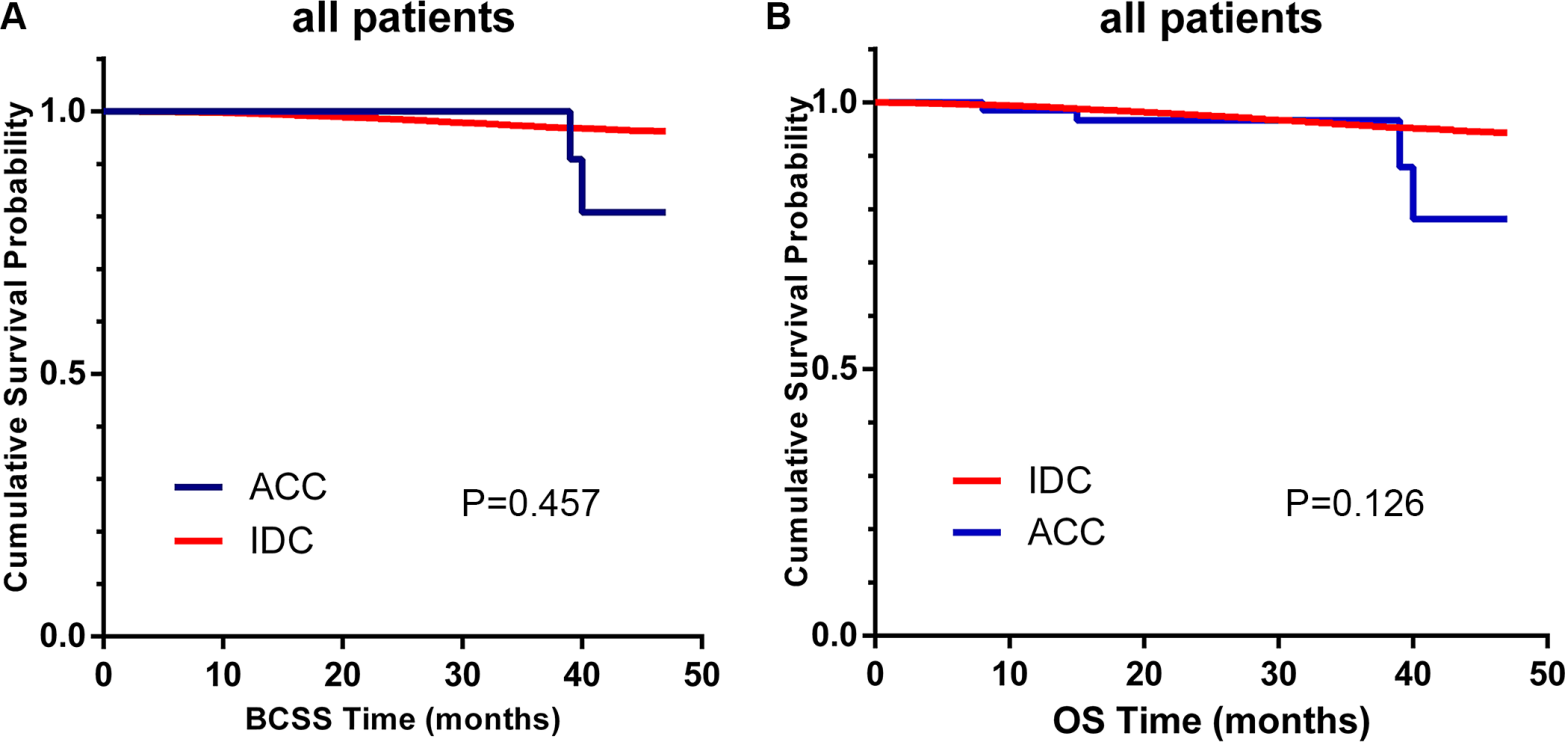
Kaplan–Meier curves of breast cancer-specific survival (BCSS, **A**) and overall survival (OS, **B**) based on histology for all patients, adenoid cystic carcinoma (ACC) vs. invasive ductal carcinoma (IDC).

Figure 1 presents the survival curves of the two histological types for breast cancer-specific survival (BCSS) and overall survival (OS) depicted using the Kaplan-Meier method. BCSS (*P* = 0.457) and OS (*P* = 0.126) were similar for these two histological types. Supplementary Table S1 and Table 2 summarize the prognostic factors according to the BCSS and OS results from univariate and multivariate Cox proportional hazard regression models, respectively. Many factors, including being black or unmarried, poor differentiation, advance stage, larger tumor size, increased number of lymph nodes, subtype of TNBC, mastectomy treatment and no radiation therapy, were significantly associated with poor BCSS and OS in univariate analysis. However, after adjusting for other prognostic factors via multivariate analysis, AJCC stage and type of surgery were no longer independent prognostic factors for OS. In addition, breast-ACC patients exhibited similar BCSS (univariate: hazard ratio (HR) = 1.357, 95% confidence interval, CI: 0.339–5.430, *P* = 0.666; multivariate: HR = 1.757, 95% CI: 0.437–7.060, *P* = 0.427) and OS (univariate: HR = 1.216, 95% CI: 0.456–3.241, *P* = 0.696, multivariate: HR = 1.127 (0.422–3.014), 95% CI: 0.422–3.014, *P* = 0.811) compared to IDC patients after univariate and multivariate analysis.

**Table 2:** Multivariate cox proportional hazard model of breast cancer-specific survival (BCSS) and overall survival (OS)

Variables		BCSS		OS	
		HRs (95% CI)	*P*^c^	HRs (95% CI)	*P*^c^
**Age (years)**	**20–49**	0.778 (0.693–0.873)	**< 0.001**	0.605 (0.547–0.669)	**< 0.001**
	**50–79**	Reference		Reference	
**Race**	**White**	Reference		Reference	
	**Black**	1.232 (1.077–1.408)	**0.002**	1.218 (1.090–1.360)	**< 0.001**
	**Other^a^**	0.675 (0.542–0.840)	**< 0.001**	0.723 (0.610–0.857)	**< 0.001**
**Marital status**	**Married**	Reference		Reference	
	**Not married^b^**	1.254 (1.125–1.397)	**< 0.001**	1.500 (1.377–1.634)	**< 0.001**
**Grade**	**I**	0.337 (0.226–0.502)	**< 0.001**	0.884 (0.747–1.046)	0.150
	**II**	Reference		Reference	
	**III and IV**	2.230 (1.926–2.581)	**< 0.001**	1.612 (1.447–1.795)	**< 0.001**
**Histology type**	**ACC**	2.548 (0.633–10.253)	0.188	2.225 (0.830–5.966)	0.112
	**IDC**	Reference		Reference	
**AJCC stage**	**I**	Reference		Reference	
	**II**	1.542 (1.200–1.980)	**0.001**	1.115 (0.924–1.344)	0.256
	**III**	1.740 (1.180–2.565)	**0.005**	1.199 (0.863–1.667)	0.280
**Tumor size (cm)**	**≤ 2**	Reference		Reference	
	**> 2 and ≤ 5**	1.655 (1.371–1.998)	**< 0.001**	1.571 (1.346–1.834)	**< 0.001**
	**> 5**	3.135 (2.477–3.968)	**< 0.001**	2.799 (2.285–3.428)	**< 0.001**
**Nodal status**	**0**	Reference		Reference	
	**1 to 3**	1.829 (1.562–2.141)	**< 0.001**	1.550 (1.368–1.756)	**< 0.001**
	**4 to 10**	3.316 (2.413–4.558)	**< 0.001**	2.783 (2.092–3.701)	**< 0.001**
	**> 10**	5.758 (4.231–7.835)	**< 0.001**	4.500 (3.405–5.947)	**< 0.001**
**Breast subtype**	**HR+/Her2-**	Reference		Reference	
	**HR+/Her2+**	0.620 (0.498–0.771)	**< 0.001**	0.706 (0.600–0.831)	**< 0.001**
	**HR-/Her2+**	1.204 (0.977–1.485)	0.082	1.107 (0.928–1.319)	0.259
	**Triple negative**	3.084 (2.718–3.498)	**< 0.001**	2.440 (2.196–2.710)	**< 0.001**
**Type of surgery**	**BCS**	Reference		Reference	
	**Mastectomy**	1.137 (1.005–1.286)	**0.041**	0.992 (0.900–1.094)	0.875
**Radiation**	**No**	1.492 (1.332–1.671)	**< 0.001**	1.829 (1.667–2.006)	**< 0.001**
	**Yes**	Reference		Reference	

Abbreviations: AJCC, American Joint Committee on Cancer; ACC, adenoid cystic carcinoma; IDC, invasive ductal carcinoma; Her2, human epidermal growth factor receptor 2; HR, hormone receptor; BCS, breast-conserving surgery; HRs, hazard ratios; CI, confidence interval; BCSS, breast cancer-specific survival; OS, overall survival.

^a^Other includes American Indian/Alaskan native, and Asian/Pacific Islander.

^b^Not married includes divorced, separated, single (never married), unmarried or domestic partner and widowed.

^c^*P* value was adjusted by multivariate Cox proportional hazard regression model including all factors, as categorized in Table 2, and bold type indicates significance.

### Survival estimates in matched groups

**Figure 2:**
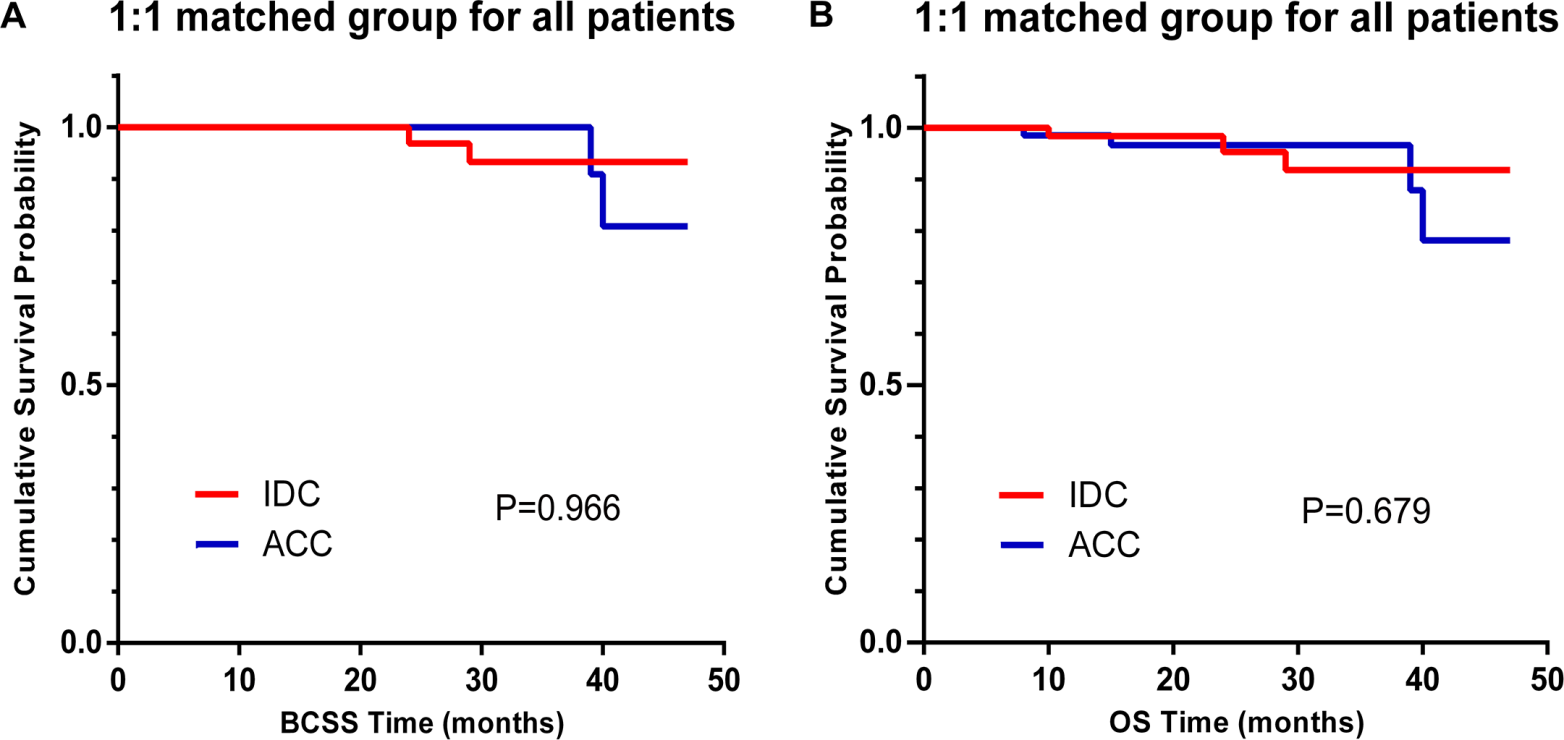
Kaplan–Meier curves of breast cancer-specific survival (BCSS, **A**) and overall survival (OS, **B**) by histology for 1:1 matched group, adenoid cystic carcinoma (ACC) vs. invasive ductal carcinoma (IDC).

We conducted 1:1 (breast-ACC/breast-IDC) matched case-control analysis using a propensity score matching method and a comprehensive consideration of the confounding factors affecting breast cancer outcomes between breast-ACC and breast-IDC patients (Table 3). Finally, we obtained a group of 172 patients, and each counterpart included 86 patients. For the matched groups, with the exception of grade and type of surgery, no factors differed significantly between breast-ACC and breast-IDC. Furthermore, we validated that IDC histology has the same prognostic value for breast-ACC patients with respect to BCSS or OS (Figure 2, P = 0.966 and *P* = 0.679 for BCSS and OS, respectively).

**Table 3:** Baseline characteristics of patients with adenoid cystic carcinoma and invasive ductal carcinoma in a 1:1 matched group

Characteristics		ACC (*n* = 86)		IDC (*n* = 86)		Total (*n* = 172)		*P*^c^
		No	%	No	%	No	%	
**Median follow-up (months) (IQR)**		18 (9–31)		21 (11–31)		18 (9–32)		
**Age (years)**	**18–49**	22	25.6	22	25.6	44	25.6	1.000
	**50–79**	64	74.4	64	74.4	128	74.4	
**Race**	**White**	72	83.7	70	81.4	142	82.6	0.768
	**Black**	11	12.8	11	12.8	22	12.8	
	**Other^a^**	3	3.5	5	5.8	8	4.7	
**Marital status**	**Married**	58	67.4	59	68.6	117	68.0	0.870
	**Not married^b^**	28	32.6	27	31.4	55	32.0	
**Laterality**	**Left**	39	45.3	46	53.5	85	49.4	0.286
	**Right**	47	54.7	40	46.5	87	50.6	
**Grade**	**I**	47	54.7	5	5.8	52	30.2	**< 0.001**
	**II**	29	33.7	22	25.6	51	29.7	
	**III and IV**	10	11.6	59	68.6	69	40.1	
**AJCC stage**	**I**	48	55.8	49	57.0	97	56.4	0.878
	**II**	38	44.2	37	43.0	75	43.6	
	**III**	0	0.0	0	0.0	0	0.0	
**Tumor size (cm)**	**≤ 2**	49	57.0	49	57.0	98	57.0	1.000
	> **2 and ≤ 5**	35	40.7	35	40.7	70	40.7	
	**> 5**	2	2.3	2	2.3	4	2.3	
**Nodal status**	**0**	84	97.7	84	97.7	168	97.7	1.000
	**1 to 3**	2	2.3	2	2.3	4	2.3	
	**4 to 10**	–	–	–	–	–	–	
	**> 10**	–	–	–	–	–	–	
**Breast subtype**	**HR+/Her2-**	17	19.8	17	19.8	34	19.8	1.000
	**HR+/Her2+**	–	–	–	–	–	–	
	**HR-/Her2+**	2	2.3	2	2.3	4	2.3	
	**Triple negative**	67	77.9	67	77.9	134	77.9	
**Type of surgery**	**BCS**	67	77.9	54	62.8	121	70.3	**0.030**
	**Mastectomy**	19	22.1	32	37.2	51	29.7	
**Radiation**	**No**	35	40.7	37	43.0	72	41.9	0.757
	**Yes**	51	59.3	49	57.0	100	58.1	

Abbreviations: ACC, Adenoid cystic carcinoma; IDC, invasive ductal carcinoma; AJCC, American Joint Committee on Cancer; Her2, human epidermal growth factor receptor 2; HR, hormone receptor; BCS, breast-conserving surgery; IQR, interquartile range.

^a^Other includes American Indian/Alaskan native and Asian/Pacific Islander.

^b^Not married includes divorced, separated, single (never married), unmarried or domestic partner, and widowed.

^c^*P* value was calculated among all groups by the Chi-square test after matching for age, tumor size, nodal status, and breast subtype, and bold type indicates significance.

### Baseline characteristics and survival outcomes in the triple-negative subgroup

**Figure 3:**
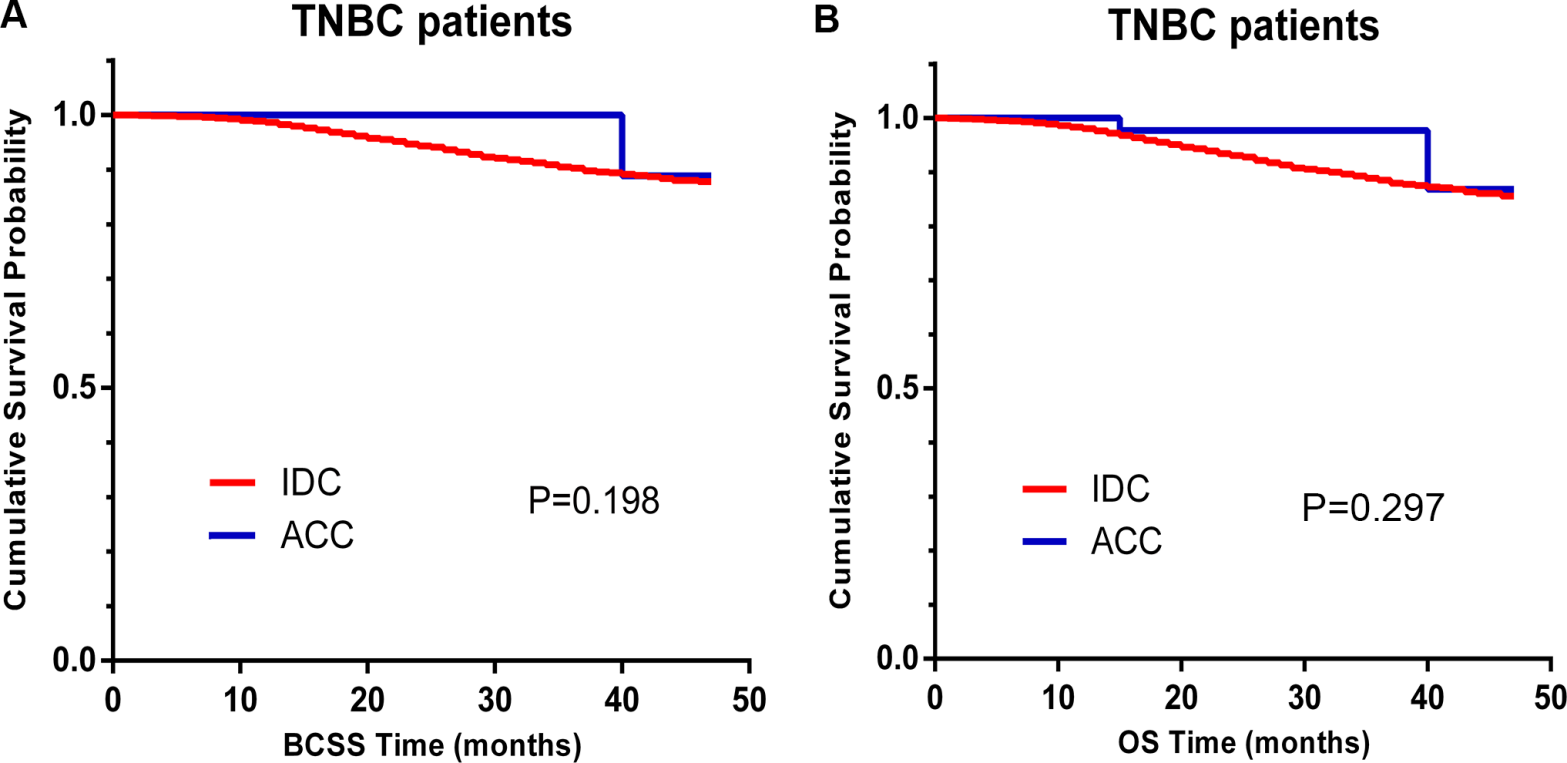
Kaplan–Meier curves of breast cancer-specific survival (BCSS, **A**) and overall survival (OS, **B**) based on histology for triple-negative breast cancer (TNBC) patients, adenoid cystic carcinoma (ACC) vs. invasive ductal carcinoma (IDC).

**Figure 4:**
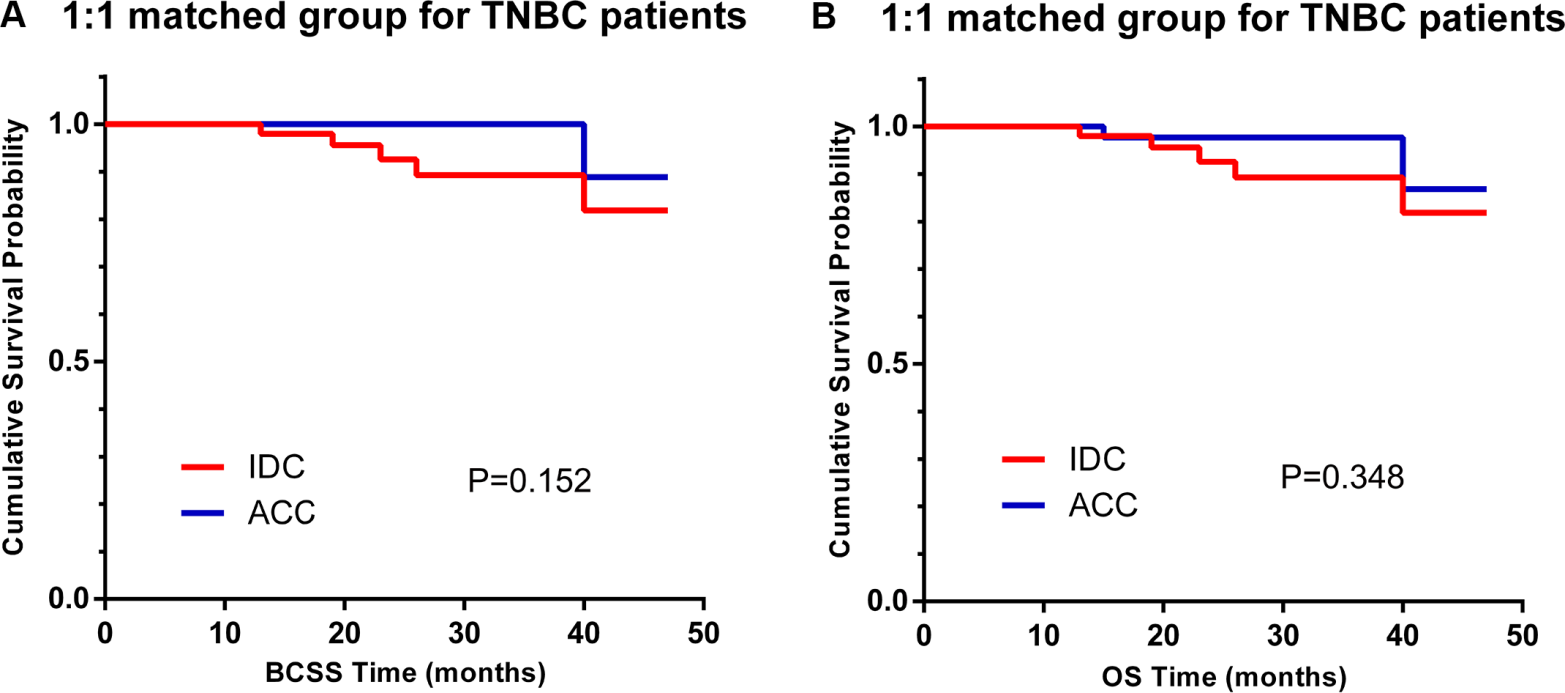
Kaplan–Meier curves of breast cancer-specific survival (BCSS, **A**) and overall survival (OS, **B**) based on histology for 1:1 matched triple-negative breast cancer (TNBC) patients, adenoid cystic carcinoma (ACC) vs. invasive ductal carcinoma (IDC).

Overall, 77.9% of breast-ACC patients were diagnosed with TNBC. To obtain deeper insight into the breast-ACC cases, we further investigated the characteristics and survival outcomes of the patients in the TNBC subgroup, which included 67 ACC patients and 11,534 IDC patients (Supplementary Table S2). When the entire population was considered, TNBC-ACC patients tended to be well-differentiated (grade I, 52.2% vs. 1.4%, *P* < 0.001), to be at an earlier stage (AJCC stage III, 0.0% vs. 11.6%, *P* = 0.005), to have a lower likelihood for nodal involvement (98.9% vs. 68.7%, *P* < 0.001), and to receive BCS (76.1% vs. 54.7%, *P* = 0.001). Kaplan-Meier curves revealed similar BCSS and OS for TNBC-ACC patients and TNBC-IDC patients (Figure 3, P = 0.198 and *P* = 0.297 for BCSS and OS, respectively). Furthermore, there were no significant differences in BCSS and OS for the 67 TNBC-ACC patients and 67 TNBC-IDC patients matched using the propensity score matching method (Figure 4, P = 0.152 and *P* = 0.348 for BCSS and OS, respectively).

### Stratification analysis with molecular subtype

We stratified molecular subtype to further validate the different outcomes affected by molecular subtype between breast-ACC and breast-IDC cases. As shown in Table 4, multivariate analysis revealed excellent survival for HR+/Her2- patients with breast-ACC compared to HR+/Her2- patients with breast-IDC according to BCSS (HR: 35.244 95% CI: 4.912–252.871, *P* < 0.001) and OS (HR: 16.137, 95% CI: 4.020–64.776, *P* < 0.001) for breast-ACC patients. However, survival was similar for ACC-TNBC and IDC-TNBC patients. These findings suggest that we can not neglect this molecular subtype when formulating therapies for breast-ACC patients.

**Table 4:** Comparison of breast cancer-specific survival (BCSS) and overall survival (OS) between adenoid cystic carcinoma and invasive ductal carcinoma after subgroup analyses using a multivariate Cox proportional hazard model

Subtype	BCSS			OS		
	Events No	HRs (95% CI)	*P*^a^	Events No	HRs (95% CI)	*P*^a^
**HR+/Her2-**			< 0.001			**< 0.001**
**ACC (*n* = 17)**	1	35.244 (4.912–252.871)		2	16.137 (4.020–64.776)	
**IDC (*n* = 63,667)**	546	Reference		1,089	Reference	
**HR+/Her2+**			–			–
**ACC (*n* = 0)**	0	–		0	–	
**IDC (*n* = 10,271)**	97	Reference		174	Reference	
**HR-/Her2+**			–		–	–
**ACC (*n* = 2)**	0	–		0	–	
**IDC (*n* = 4,379)**	111	Reference		152	Reference	
**Triple negative**			0.868			0.603
**ACC (*n* = 67)**	1	0.845 (0.115–6.189)		2	1.456 (0.353–6.000)	
**IDC (*n* = 11,534)**	634	Reference		773	Reference	

Abbreviation: ACC, Adenoid cystic carcinoma; IDC, invasive ductal carcinoma; HR, hormone receptor; Her2, human epidermal growth factor receptor 2; HR, hazard ratio; CI, confidence interval; BCSS, breast cancer-specific survival; OS, overall survival.

^a^*P* value was adjusted by a multivariate Cox proportional hazard regression model including age, race, marital status, grade, AJCC stage, tumor size, lymph node status, type of surgery, and radiation, and bold type indicates significance.

## DISCUSSION

Using this large amount of population-based data, we aimed to analyze the characteristics and outcomes of breast-ACC patients compared to breast-IDC patients. Our findings indicate that breast-ACC has distinct clinical and pathological characteristics and exhibits an indolent clinical course compared to breast-IDC. However, we did not observe improved survival for breast-ACC compared to breast-IDC after adjusting and matching confounding factors. Moreover, further subgroup analysis of molecular subtypes revealed improved survival in breast-ACC patients with HR+/Her2- compared to breast-IDC patients with HR+/Her2-, whereas survival was similar for ACC-TNBC and IDC-TNBC.

Kulkarni et al. [9] compared breast-ACC and breast-IDC using national cancer data and observed distinct differences in median tumor size, histological grade, node positive rate, BCS type, and hormone therapy between the two tumor types. Slightly larger tumor size, more cases of grade I, a lower rate of node positivity, more BCS and less hormone therapy were observed for breast-ACC. Similarly, in our study, breast-ACC patients presented with a higher proportion of TNBC and were more likely to have well-differentiated tumors, a less advanced stage, and rare regional lymph node involvement; compared to the breast-IDC group, more of these patients received BCS. Accordingly, we inferred that breast-ACC patients have unique clinical and pathological characteristics and that the disease was inclined to more indolent behavior in these patients.

Favorable prognosis for breast-ACC has already been demonstrated [18]. Although there was no significant difference in survival for grade 1 and stage 1 patients when comparing breast-ACC and breast-IDC, Kulkarni et al. [9] observed better 5-year overall survival in breast-ACC when compared to breast-IDC in the entire cohort, indicating that the increased OS may largely be explained by the lower grade and earlier stage of patients presenting with the former compared to the latter. However, in our study, the histology type was not a surrogate for better survival in breast-ACC and breast-IDC patients. Instead, older age, black race, unmarried, higher grade, larger tumor size, a more positive nodal status, TNBC subtype and no radiation therapy were significantly associated with poor BCSS and OS.

Although most breast-ACC cases are HR-, rare HR+ cases have been reported [8–10]. When referring to molecular subtype, no complete information in a related large population-based study has been observed. To further investigate probable prognostic factors, we conducted a short-term survival comparison between breast-ACC and breast-IDC patients based on molecular subtype. To our surprise, our results differed from those of previous studies. We observed similar survival among patients with breast-ACC compared to breast-IDC, and this result was validated by a propensity score matching method. Additionally, BCSS and OS did not differ significantly between TNBC-ACC and TNBC-IDC before and after matching based on age, tumor size, and nodal status. Subgroup analyses revealed similar survival for ACC-TNBC and IDC-TNBC; however, excellent survival was observed in HR+/Her2- breast-ACC patients compared to HR+/Her2- patients with breast-IDC. Accordingly, we recognized that the distinct prognostic outcomes are driven in part by the molecular subtype of breast cancer.

Compared to other studies of breast-ACC, our investigation has two major advantages. First, we used HR and HER2 information and demonstrated survival outcomes in detail according to the molecular subtype in a related large population dataset. Second, we conducted propensity score matching to diminish the effects of confounding factors, which guaranteed more persuasive statistical analyses. Unfortunately, due to the limitations of the SEER database, we did not have information on Ki-67 expression to further subdivide the molecular subtype. Additionally, information on adjuvant chemotherapy and endocrine therapy was not available for our study, which may conceal important prognostic factors affecting the outcomes of cancer. Due to the lack of HER2 status information before 2010, we focused on short-term survival, and the inadequate follow-up time may have biased the results. However, for the TNBC subtype, an early peak of recurrence occurs within the first 2–3 years after diagnosis [19].

In summary, understanding clinical characteristics and outcomes can provide doctors with evidence to support the same intensive treatment and attention for ACC-TNBC as IDC-TNBC and might lead to more individualized and tailored therapy for breast-ACC patients. However, further subdivision of molecular subtype based on Ki67 expression is needed to validate this conclusion.

## MATERIALS AND METHODS

### Ethics statement

Because cancer is a reportable disease in every state in the United States, we did not need to obtain patient consent but were required to sign a Data-Use Agreement for the SEER 1973-2013 Research Data File to gain access to the SEER database.

### Patients

We used SEER*Stat version 8.3.2. to extract data from the SEER 18 registries research database, including data from 1973 to 2013, and our results generated a case listing with a total of 89,937 eligible patients, including 86 breast-ACC patients and 89,851 breast-IDC patients. In this study, we examined cases of female breast cancer diagnosed with histologically confirmed first invasive breast cancer according to the following criteria: year of diagnosis from 2010 to 2013, age at diagnosis between 20 and 79 years, race, marital status at diagnosis, breast cancer as the first and only malignant cancer, pathologically confirmed ACC (ICD-O-3 8200/3) or IDC-NOS (ICD-O-3 8500/3), unilateral origin of primary cancer, histological grades I to IV, TNM stages I-III, known ER, PR and HER2 status, breast subtype, surgery treatment with either mastectomy or BCS, record of radiation therapy, cause of death, and survival (months). Tumors of any size with direct extension to the chest wall and/or to the skin (T4, including ulceration, skin nodules and inflammatory carcinoma) were not included in the study. To obtain data on HER2 status and ensure adequate follow-up duration, we calculated follow-up times from January 1, 2010, to December 31, 2013.

### Outcome measurement

We defined BCSS as the date of diagnosis to the date of death due to breast cancer, and OS was calculated from the date of diagnosis to the date of death regardless of whether the death was related to breast cancer. Patients who were alive were censored on the date of last contact for both outcomes.

### Statistical analysis

The chi-square test was employed to describe the demographic and clinical characteristics of the breast-ACC and breast-IDC groups, including the whole group and 1:1 matched group as well as the TNBC group. The Kaplan-Meier method was used to generate survival curves, and the log-rank test was performed to determine whether the differences in BCSS or OS rates between different histological subtypes were statistically significant. A Cox proportional hazards model was utilized to calculate the HR ratio and 95% confidence intervals in the univariate and multivariate analyses and to identify prognostic factors. These statistical analyses were conducted using SPSS version 21.0. To diminish the effects of baseline differences in demographic and clinical characteristics across histology subtypes for outcome differences, we applied the psmatch2 module to perform propensity score matching [20] in Stata version 14.0. The command matched each breast-ACC patient to one breast-IDC patient using the following factors: age, tumor size, nodal status, and breast subtype. All *P* values were two-sided, and values less than 0.05 were considered statistically significant.

## SUPPLEMENTARY MATERIALS TABLES


