# Supplementary material for: Similar outcomes between adenoid cystic carcinoma of the breast and invasive ductal carcinoma: a population-based study from the SEER 18 database
Source: Oncotarget. 2016 Dec 20;8(4):6206–15. doi: 10.18632/oncotarget.14052 (PMC5351624; doi:10.18632/oncotarget.14052)
Supplement: Supplementary file 1 [file oncotarget-08-6206-s001.pdf]

Similar outcomes between adenoid cystic carcinoma of the breast and invasive ductal carcinoma: a population-based study from the SEER 18 database

Supplementary Materials

Supplementary Table S1: Univariate cox proportional hazard model of breast cancer-specific survival (BCSS) and overall survival (OS)

Variables		BCSS		OS	
		HRs (95% CI)	*P*	HRs (95% CI)	*P*
**Age (years)**	**20–49**	1.251 (1.117–1.401)	**< 0.001**	0.835 (0.757–0.922)	**< 0.001**
	**50–79**	Reference		Reference	
**Race**	**White**	Reference		Reference	
	**Black**	2.175 (1.911–2.476)	**< 0.001**	1.918 (1.723–2.135)	**< 0.001**
	**Other[a]**	0.681 (0.547–0.848)	**0.001**	0.708 (0.598–0.838)	**< 0.001**
**Marital status**	**Married**	Reference		Reference	
	**Not married[b]**	1.497 (1.348–1.664)	**< 0.001**	1.743 (1.603–1.896)	**< 0.001**
**Laterality**	**Left**	Reference		Reference	
	**Right**	0.941 (0.847–1.046)	0.261	0.933 (0.858–1.014)	0.104
**Grade**	**I**	0.196 (0.132–0.292)	**< 0.001**	0.624 (0.530–0.736)	**< 0.001**
	**II**	Reference		Reference	
	**III and IV**	4.591 (4.017–5.247)	**< 0.001**	2.716 (2.467–2.990)	**< 0.001**
**Histology type**	**ACC**	1.683 (0.420–6.735)	0.462	2.114(0.793–5.636)	0.135
	**IDC**	Reference		Reference	
**AJCC stage**	**I**	Reference		Reference	
	**II**	5.074 (4.323–5.955)	**< 0.001**	2.609 (2.353–2.893)	**< 0.001**
	**III**	17.866 (15.193–21.010)	**< 0.001**	7.092 (6.344–7.927)	**< 0.001**
**Tumor size (cm)**	**≤ 2**	Reference		Reference	
	**> 2 and ≤ 5**	4.845 (4.260–5.510)	**< 0.001**	2.873 (2.620–3.150)	**< 0.001**
	**> 5**	13.723 (11.747–16.030)	**< 0.001**	6.603 (5.819–7.493)	**< 0.001**
**Nodal status**	**0**	Reference		Reference	
	**1 to 3**	2.949 (2.592–3.356)	**< 0.001**	1.955 (1.770–2.159)	**< 0.001**
	**4 to 10**	7.013 (5.999–8.198)	**< 0.001**	4.106 (3.606–4.675)	**< 0.001**
	**> 10**	15.537 (13.275–18.184)	**< 0.001**	8.186 (7.143–9.381)	**< 0.001**
**Breast subtype**	**HR+/Her2-**	Reference		Reference	
	**HR+/Her2+**	1.108 (0.893–1.375)	0.351	0.996 (0.849–1.169)	0.966
	**HR-/Her2+**	2.881 (2.349–3.533)	**< 0.001**	1.980 (1.671–2.346)	**< 0.001**
	**Triple negative**	6.312 (5.630–7.077)	**< 0.001**	3.860 (3.520–4.232)	**< 0.001**
**Type of surgery**	**BCS**	Reference		Reference	
	**Mastectomy**	2.677 (2.401–2.985)	**< 0.001**	2.019 (1.856–2.197)	**< 0.001**
**Radiation**	**No**	1.480 (1.332–1.644)	**< 0.001**	1.780 (1.637–1.936)	**< 0.001**
	**Yes**	Reference		Reference	

Abbreviations: AJCC, American Joint Committee on Cancer; ACC, adenoid cystic carcinoma; IDC, invasive ductal carcinoma; Her2, human epidermal growth factor receptor 2; HR, hormone receptor; BCS, breast-conserving surgery; HRs, hazard ratios; CI, confidence interval; BCSS, breast cancer-specific survival; OS, overall survival.

[a]Other includes American Indian/Alaskan native and Asian/Pacific Islander.

[b]Not married includes divorced, separated, single (never married), unmarried or domestic partner and widowed.

Supplementary Table S2: Baseline characteristics of patients with triple negative tumors, ACC vs. IDC

Characteristics		ACC (*n* = 67)		IDC (*n* = 11,534)		Total (*n* = 11,601)		*P*[c]
		No	%	No	%	No	%	
**Age (years)**	**20–49**	20	29.9	3,769	32.7	3,789	32.7	0.623
	**50–79**	47	70.1	7,765	67.3	7,812	67.3	
**Race**	**White**	56	83.6	8,366	72.5	8,422	72.6	0.112
	**Black**	9	13.4	2,297	19.9	2,306	19.9	
	**Other**[a]	2	3.0	871	7.6	873	7.5	
**Marital status**	**Married**	43	64.2	7,077	61.4	7,120	61.4	0.636
	**Not married**[b]	24	35.8	4,457	38.6	4,481	38.6	
**Laterality**	**Left**	27	40.3	5,938	51.5	5,965	51.4	0.068
	**Right**	40	59.7	5,596	48.5	5,636	48.6	
**Grade**	**I**	35	52.2	163	1.4	198	1.7	**< 0.001**
	**II**	25	37.3	1,795	15.6	1,820	15.7	
	**III and IV**	7	10.4	9,576	83.0	9,583	82.6	
**AJCC stage**	**I**	36	53.7	4,708	40.8	4,744	40.9	**0.005**
	**II**	31	46.3	5,493	47.6	5,524	47.6	
	**III**	0	0.0	1,333	11.6	1,333	11.5	
**Tumor size (cm)**	**≤2**	36	53.7	5,543	48.1	5,579	48.1	0.311
	**>2 and >5**	29	43.3	5,114	44.3	5,143	44.3	
	**>5**	2	3.0	877	7.6	879	7.6	
**Nodal status**	**0**	66	98.5	7,921	68.7	7,987	68.8	**< 0.001**
	**1 to 3**	1	1.5	2,622	22.7	2,623	22.6	
	**4 to 10**	0	0.0	617	5.3	617	5.3	
	**> 10**	0	0.0	374	3.2	374	3.2	
**Type of surgery**	**BCS**	51	76.1	6,314	54.7	6,365	54.9	**0.001**
	**Mastectomy**	16	23.9	5,220	45.3	5,236	45.1	
**Radiation**	**No**	26	38.8	5,134	44.5	5,160	44.5	0.349
	**Yes**	41	61.2	6,400	55.5	6,441	55.5	

Abbreviations: ACC, adenoid cystic carcinoma; IDC, invasive ductal carcinoma; AJCC, American Joint Committee on Cancer; BCS, breast-conserving surgery.

[a]Other includes American Indian/Alaskan native and Asian/Pacific Islander.

[b]Not married includes divorced, separated, single (never married), unmarried or domestic partner and widowed.

[c]*P* value was calculated among all groups by the Chi-square test, and bold type indicates significance.
